# In Search of an Efficient and Reliable Deep Learning Model for Identification of COVID-19 Infection from Chest X-ray Images

**DOI:** 10.3390/diagnostics13030574

**Published:** 2023-02-03

**Authors:** Abul Kalam Azad, Mahabub-A-Alahi, Imtiaz Ahmed, Mosabber Uddin Ahmed

**Affiliations:** Department of Electrical and Electronic Engineering, University of Dhaka, Dhaka 1000, Bangladesh

**Keywords:** image classification, chest X-ray images, local binary pattern, machine learning classifiers, support vector machine, pattern recognition network, convolutional neural network

## Abstract

The virus responsible for COVID-19 is mutating day by day with more infectious characteristics. With the limited healthcare resources and overburdened medical practitioners, it is almost impossible to contain this virus. The automatic identification of this viral infection from chest X-ray (CXR) images is now more demanding as it is a cheaper and less time-consuming diagnosis option. To that cause, we have applied deep learning (DL) approaches for four-class classification of CXR images comprising COVID-19, normal, lung opacity, and viral pneumonia. At first, we extracted features of CXR images by applying a local binary pattern (LBP) and pre-trained convolutional neural network (CNN). Afterwards, we utilized a pattern recognition network (PRN), support vector machine (SVM), decision tree (DT), random forest (RF), and k-nearest neighbors (KNN) classifiers on the extracted features to classify aforementioned four-class CXR images. The performances of the proposed methods have been analyzed rigorously in terms of classification performance and classification speed. Among different methods applied to the four-class test images, the best method achieved classification performances with 97.41% accuracy, 94.94% precision, 94.81% recall, 98.27% specificity, and 94.86% F1 score. The results indicate that the proposed method can offer an efficient and reliable framework for COVID-19 detection from CXR images, which could be immensely conducive to the effective diagnosis of COVID-19-infected patients.

## 1. Introduction

The severe acute respiratory syndrome coronavirus 2 (SARS-CoV-2) is responsible for the devastating coronavirus disease 2019 (COVID-19) outbreak which rapidly spread across the world and took over 6.55 million human lives to date [1,2,3]. The SARS-CoV-2 is a single-stranded and approximately spherically shaped enveloped RNA virus with probable zoonotic origin belonging to the coronaviridae family [4,5,6]. Although the SARS-CoV-2 infection can occur at all ages of human beings, its symptoms and acuteness vary with age from asymptomatic (in cases of children and young people) to severe respiratory malfunction and organ failure (above 60 y) [2,7]. The most common COVID-19 symptoms are fever, fatigue, and dry cough, whereas, headache, nausea, sore throat, sputum production, hemoptysis, diarrhea, anorexia, and chest pain are noted as less common features of it [8,9,10,11]. The gold standard of COVID-19 diagnosis is based on the detection of SARS-CoV-2 nucleic acid at a molecular level by real-time reverse transcription polymerase chain reaction (RT-PCR) [12,13]. However, the laboratory-grade accuracy and specificity of RT-PCR can be adversely affected by the variability of type, method of handling, and collection time of the sample with respect to the disease stage which may lead to a low detection rate below 50% [14,15,16,17]. As the early sign of acute COVID-19 reveals symptoms of pneumonia, radiological imaging techniques such as chest X-ray (CXR), computed tomography (CT), and magnetic resonance imaging (MRI) serve as indispensable tools to negate the false negative RT-PCR detection [18,19,20,21,22,23,24,25,26,27]. Although CXR is preferred over the sophisticated CT and MRI in terms of cost, equipment availability, measurement time, and radiation dose, the proper diagnosis of COVID-19 from it poses significant challenges in many parts of the world, arising from the scarcity of experienced radiologists for visual inspection of minute features such as ground-glass opacity and abnormalities of bilateral and interstitial nature in the radiographs relevant to COVID-19 pneumonia [9,11,23,28]. Moreover, the similarity of CXR images of other viral infections and respiratory diseases with COVID-19 makes it very challenging even for expert radiologists [8,21]. Artificial intelligence (AI)-based deep machine learning (ML) offers tremendous support in revealing subtle and hidden image features which may not be apparent in the original CXR and thereby facilitating the automation of image classification and COVID-19 disease diagnosis problem [29]. Moreover, the abundance of publicly available CXR image datasets provided tremendous facilitation in training ML algorithms [30].

Numerous attempts have been made to standardize the detection models for COVID-19 based on CXR images as a complement to RT-PCR using an AI-based DL approach [31,32,33]. The success of CXR image-based COVID-19 diagnosis with DL potentially relies on the reliable detection of different abnormalities of interstitial and bilateral nature as well as ground-glass opacity irregularities in CXR radiographs [34]. The local binary pattern (LBP) operator is a very popular feature extraction method for CXR radiographs due to its simplicity and low computational cost. The LBP operator divides the images into several parts and assigns binary patterns depending on the texture of the pixel neighbor to extract the feature. On the other hand, a sophisticated and computationally intense DL-based convolutional neural network (CNN), due to its inherent spatial feature extraction and classification capabilities, has emerged as a leading candidate for CXR-based COVID-19 detection [35,36,37,38]. With the aid of transfer learning, efficient training of deep CNN with a relatively small CXR dataset sparked its widespread usage in the ML research community to differentiate COVID-19 from non-COVID-19 pneumonia from CXR images [39].

Many of the CXR image-based detection techniques use off-the-rack deep neural networks such as VGG-16, VGG-19, Inception-V3, MobileNet-V2, ResNet-18, ResNet-50, DenseNet-121, CapsNet, and EfficientNet. The CNN network prefers large datasets to avoid bias problem and hence the size of the dataset is crucial for the reliability of result presented in the different literature. For a small dataset containing 125 CXR images, 98.08% accuracy in COVID-19 classification was reported for binary classes in Ref. [40]. Ismael et al., reported an accuracy of 92.63% in COVID-19 prediction working on a CXR image set containing 180 COVID-19 and 200 normal patients’ images, where they used a pre-trained ResNet50 model for feature extraction and support vector machine (SVM) with a linear kernel for classification [41]. Afshar et al., used similar two-class CXR images with CapsNet to obtain an accuracy of 98.3% [42]. Frid-Adar et al., achieved 98% accuracy using the ResNet50 over a two-class dataset of no lung opacity and lung opacity [43]. For a large two-class CXR dataset with COVID-19 pneumonia (5805) and non–COVID-19 pneumonia (5300) images, Zhang et al., managed to report 92% accuracy using the DenseNet-121 [44]. Vinod and Prabaharan used a pre-trained CNN in combination with a decision tree classifier to obtain a precision score of 88% on a CXR dataset containing ~300 two-class images [45].

For a three-class classification problem, the achieved accuracy was 95% over a CXR dataset containing normal (104), non-COVID-19 pneumonia (80), and COVID-19 pneumonia (99) [46]. Bukhari et al., reported overall accuracy of 98.24% using 278 images in ResNet-50 with three different image classes comprised of normal (93), COVID-19 (89), and non-COVID-19 pneumonia (96) [47]. Tartaglione et al., achieved a maximum 85% accuracy over 5857 CXR images with three classes comprising normal (1583), bacterial pneumonia (2780), and viral pneumonia (1493) [48]. For a different three-class CXR image dataset containing normal (1314), community-acquired pneumonia (2004), and COVID-19 pneumonia (204), the reported maximum accuracy was 98.71% [49]. Kana et al., obtained 99% accuracy for a dataset with normal (2487), bacterial/viral pneumonia (2507), and COVID-19 (161) [50]. On a similar three-class problem, 91.92% overall accuracy of prediction was achieved in Ref. [44]. Bassi and Attux reported an accuracy of 99.94% for a small dataset with 439 images [51]. The VGG-19 produced an accuracy of 89.3% for a dataset having normal (300), pneumonia (30), and COVID-19 (260) CXR images [52]. Luz et al., performed classification with EfficientNet on a dataset containing 13,569 CXR images and obtained 93.9% accuracy [53]. The use of a deep CNN DeTraC produced 93.1% accuracy in COVID-19 detection using a small dataset composed of normal (80), SARS (11), and COVID-19 (105) CXR images [54]. Another variant of deep CNN, known as COVID-Net achieved a prediction accuracy of 83.5% on a larger dataset containing normal (358), non-COVID-19 pneumonia (8066), and COVID-19 pneumonia (5538) CXR images [22]. The deep Bayes-SqueezeNet was capable of producing 98.3% COVID-19 prediction accuracy which utilized a dataset with normal (1583), non-COVID-19 pneumonia (4290), and COVID-19 (76) CXR images [55]. Heidari et al., used three classes of CXR images with normal (2880), non-COVID-19 pneumonia (5179), and COVID-19 pneumonia (415) to obtain an accuracy of 94.5% [56]. The classification accuracy can be increased to 97.8% with the VGG-16 CNN model with a similar-sized dataset [57]. Vinod et al., used Covix-Net to yield 96.8% accuracy on a three-class CXR database containing a total of 9000 images divided into normal (3000), COVID-19 (3000), and pneumonia (3000) [58].

In recent times, few investigations emerged with four-class classification capabilities in COVID-19 identification from CXR images. Hussain et al., used a small CXR dataset with normal (138), bacterial pneumonia (145), non-COVID-19 viral pneumonia (145), and COVID-19 (130) and managed to produce 79.52% accuracy [59]. The deep CNN termed as CoroNet was able to produce 89.6% over a CXR dataset composed of normal (310), bacterial pneumonia (330), viral pneumonia (327), and COVID-19 (284) [60]. The use of deep ResNet improved the accuracy of the four-class classification to 92.1% for a very small dataset consisting of 450 CXR images [61]. Attaullah et al., combined patients’ symptoms with a total of 800 four-class CXR images and obtained an accuracy of 78.88% [62]. The estimation of the uncertainty in the deep CNN with a Bayesian approach can improve the reliability of the accuracy measurements in a four-class classification problem [63]. The pre-trained and fine-tuned ResNet-50 architecture have been shown to achieve 96.23% accuracy for four-class CXR dataset containing normal (1203), non-COVID-19 viral pneumonia (660), bacterial pneumonia (931), and COVID-19 pneumonia (68) [64]. It is hard to find rigorous and extensive studies of COVID-19 identification within the framework of four-class classification problems using different ML algorithms on a relatively large dataset in the existing literature.

To that cause, here we used different ML algorithms on a comparatively large dataset comprising of 5360 CXR images containing four different classes, i.e., COVID-19, normal, lung opacity, and viral pneumonia, each of which contains 1340 images. The CXR image feature extractions were performed using local binary pattern (LBP) and pre-trained CNN. We used LBP-based PRN, LBP-based SVM, LBP-based DT, LBP-based RF, LBP-based KNN, and CNN-based SVM for image class identification. For the reliable performance analysis of LBP-PRN, a variety of six different training algorithms were used. The performance of SVM classifiers was assessed for nineteen different pre-trained CNNs in feature extraction. The classification performance provided by the ensemble configuration of the three best CNN-based SVM classifiers selected from these aforementioned nineteen different CNNs was also evaluated in this study. Overall, we believe that the results presented here have established an efficient and reliable CNN-based SVM framework for COVID-19 detection from CXR images.

## 2. Materials and Methods

**Figure 1:**
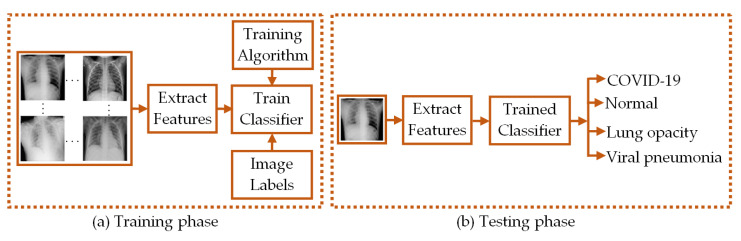
Functional diagram depicting the (**a**) training and (**b**) testing phases of classifiers.

Here, we classified four-class CXR images of COVID-19 infected patients (COVID-19 class), healthy persons (normal class), persons with lung opacity (lung opacity class), and viral pneumonia infected patients (viral pneumonia class) using LBP- and CNN-based feature extraction from the CXR images. The LBP-extracted features were subsequently used to train the PRN, SVM, DT, RF, and KNN-based machine learning classifiers. Moreover, the pre-trained CNN-derived features were subjected to SVM-based classification. The functional diagram of such feature extraction-based classifiers is depicted in Figure 1.

In both training and testing phases of the classification process, feature extraction algorithms were used to provide the necessary CXR image features. The classifiers are trained with the features obtained in the training phase and the trained classifiers are used to classify the CXR images based on the image features obtained in the testing phase.

### 2.1. Dataset of CXR Images

The dataset of CXR images used in this study has been collected from a public source [65]. Table 1 outlines a brief description of the number of CXR images in the dataset along with the number of images used in this study.

**Figure 2:**
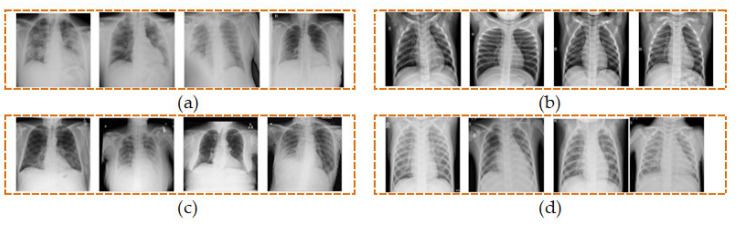
Samples of CXR images from four classes of (**a**) COVID-19, (**b**) normal, (**c**) lung opacity, and (**d**) viral pneumonia.

We have utilized a total of 5360 CXR images from the four different classes comprising of COVID-19, normal, lung opacity, and viral pneumonia, each of which contains an equal number of 1340 images as shown in Table 1. Four sample CXR images from each class are shown in Figure 2.

### 2.2. Extraction of Features from CXR Images

The feature extraction process maps the most significant information of image to a much reduced-size feature vector. In this work, we have demonstrated the use of LBP operator and pre-trained CNN to extract features from CXR images.

#### 2.2.1. Extraction of Features from CXR Images Using LBP Operator

The extraction of image features using LBP operator has found extensive applications in the field of image processing [66,67,68,69]. The basic principle of LBP operator was first presented in Ref. [66] to describe the texture of an image. This operator works by thresholding the gray levels of neighborhood pixels compared to that of their central pixel in a local circular region. The thresholded values are then summed up in a clockwise direction after being weighted by powers of 2 to obtain the gray levels of the central pixel. The LBP value of a given pixel is given by [66]:

(1) $$L_{P,R}\left(u,v\right)=\overset{P-1}{\underset{m=0}{\sum }}F\left(g_{m}-g_{c}\right)2^{m},\mathrm{where}F\left(x\right)=\left\{\begin{array}{cc} 1 & x\geq 0\\ 0 & x<0 \end{array}\right.$$

In Equation (1), *P* is the total number of neighborhoods of the central pixel in a region of the image having radius *R*, *g_m_* stands for gray level of the neighborhood pixel and *g_c_* represents the gray level of the central pixel within the region considered in the image.

**Figure 3:**
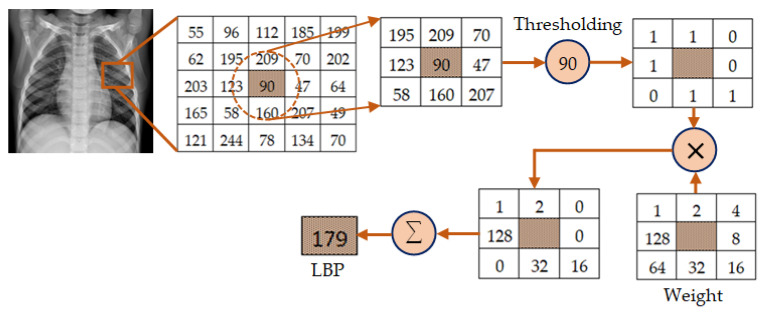
Illustration of the mechanism of feature extraction using LBP for *P* = 8 and *R* = 1.

The mechanism of feature extraction using LBP is depicted in Figure 3. In this illustration, a local region having radius *R* = 1 is considered to attain an image segment of 3×3 pixels. There are 8 pixels (*P* = 8) in this region excluding the central pixels. Then, the thresholding operation is performed through which the pixel value is set to 0 if the gray level of any neighboring pixel is less than that of the center pixel which is 90 in this illustration. Otherwise, it is set to 1. The binary values obtained through this thresholding operation are then weighted by powers of 2 sequentially in a clockwise fashion. These weighted values are finally summed up to obtain the LBP value of the central pixel. The process is repeated to obtain the LBP values of central pixels of other local regions in the whole image.

For an M × N image, a total of 2*^P^* local binary patterns *L_P,R_* are obtained from Equation (1) which are then represented as a histogram vector *I* of length 0 ≤ *l*≤ (2*^P^* − 1) as given by

(2) $$I\left[l\right]=\frac{\overset{M-1}{\underset{u=0}{\sum }}\overset{N-1}{\underset{v=0}{\sum }}L_{P,R}\left(u,v\right)}{M\times N}$$

The *L_P,R_* operator given by Equation (1) generates 2*^P^* different local binary patterns. These local binary patterns vary in accordance with the rotation of image. To avoid the effect of this rotation, the rotation invariant uniform (riu) LBP operator is used in this study, which is defined by [66]:

(3) $$L_{P,R}^{riu2}=\overset{P-1}{\underset{m=0}{\sum }}F\left(g_{m}-g_{c}\right)=\left\{\begin{array}{cc} \overset{P-1}{\underset{m=0}{\sum }}F\left(g_{m}-g_{c}\right), & U\left(L_{P,R}\right)\leq 2\\ P+1 & \mathrm{otherwise} \end{array}\right.$$

where *U*(*L_P,R_*) is a measure of uniformity, which corresponds to the number of bitwise transitions from 0 to 1 or 1 to 0 in *L_P,R_*. The superscript 2 on the left side of Equation (3) signifies the utilization of riu patterns having *U* value of no more than 2. All non-uniform *L_P,R_* obtained through Equation (1) are now grouped as one pattern. As a result, the use of Equation (3) gives a total of *P* + 2 different riu LBP [66,69]. In our study, we have considered *R* = 1, for which *P* = 8. As a result, the length of the histogram vector (i.e., length of the feature vector) for each CXR image is only 10.

#### 2.2.2. Extraction of Features from CXR Images Using CNN

**Figure 4:**
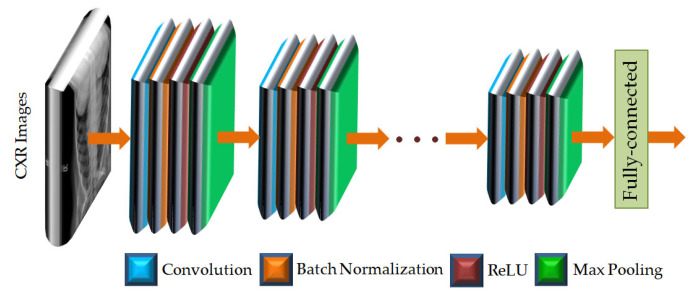
The layered architecture of a general CNN.

The convolutional neural network (CNN) is a dominant tool widely used for extracting features from images using deep learning algorithms [30,31,32,33,34]. A CNN can effectively extract the spatial and temporal characteristics in an image by utilizing shared weight structure of convolution filters to provide crucial features of an image. The CNN architecture consists of a large number of convolutional layers, batch normalization layers, rectified linear units (ReLU), and pooling layers [30,39]. The organizations of such architecture are different for different pre-trained CNNs. The layered architecture of a general CNN is given in Figure 4.

As shown in Figure 4, the CXR images are applied to the input layer of the CNN. The image input layer is followed by repeated and sequential arrangement of convolutional layers, batch normalization layers, rectified linear units (ReLUs), and max pooling layers. In each of the convolutional layers, padding is added to the input feature map for ensuring the output size is equal to the input size. In the layered arrangement, the convolutional layer is followed by the batch normalization layer to normalize the activations and gradients propagating through the CNN. The ReLU is then used to perform the process of nonlinear activation. Such ReLU layers also help to speed up the training of the CNN and reduce the sensitivity to the parameter initialization. Next, the max pooling layer is used, in which the function is to perform down-sampling so as to reduce the size of the feature map as well as to eliminate redundant information. The features of the CXR images are provided by the fully connected layer at the end of the CNN as feature vectors. The CNNs used in this study are listed in Table 2 along with the fully connected feature layer from which feature vectors have been extracted.

### 2.3. Classification of CXR Images

In this study, the classification of CXR images has been performed based on the extracted features of CXR images. For such classification, we have utilized several widely used classifiers, i.e., PRN, SVM, DT, RF, and KNN.

#### 2.3.1. Classification of CXR Images Using PRN Classifier

**Figure 5:**
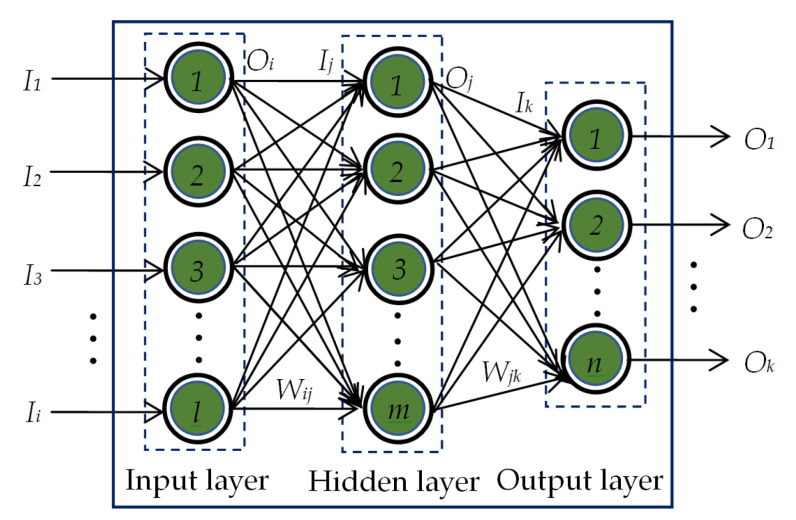
The basic architecture of a single-hidden-layer PRN.

The pattern recognition network (PRN) used in this study is a feedforward neural network comprising the input layer, a single hidden layer and the output layer. The PRN has been trained first by using known input-output patterns to optimize the weights of interconnection between the neurons in its different layers. The basic architecture of the PRN used is shown in Figure 5.

The output of the *i*th neuron in the input layer equals to the input to the *i*th neuron of the same input layer, i.e.,

(4) $$O_{i}=I_{i},i=1,2,3,\ldots ,l$$

The input *I_j_* to the *j*th neuron in the hidden layer is calculated to be the weighted sum of *O_i_* and *W_ij_*, where *W_ij_* is the weight connecting *i*th neuron in the input layer and *j*th neuron in the hidden layer. This *I_j_* is then passed through the activation function. In this work, hyperbolic tangent sigmoid function is used for the neurons in the hidden layer. Thus, the output of *j*th neuron in the hidden layer yields

(5) $$O_{j}=\frac{2}{1+e^{-2I_{j}}}-1,\mathrm{where}I_{j}=\overset{l}{\underset{i=1}{\sum }}\left(W_{ij}O_{i}\right)\mathrm{and}j=1,2,3,\ldots ,m$$

Similarly, the input *I_k_* to the *k*th neuron in the output layer is calculated to be the weighted sum of *O_j_* and *W_jk_*, where *W_jk_* is the weight connecting *j*th neuron in the hidden layer and *k*th neuron in the output layer. Initially, the output of the *k*th neuron in the output layer is computed by

(6) $$O_{k}=\frac{e^{I_{k}}}{\overset{n}{\underset{k=1}{\sum }}e^{I_{k}}},\mathrm{where}I_{k}=\overset{m}{\underset{j=1}{\sum }}\left(W_{jk}O_{j}\right)\mathrm{and}k=1,2,3,\ldots ,n$$

This *O_k_* is then passed through the softmax activation function. For using this activation function, the output of a particular neuron in the output layer is assumed to be 1 if its value calculated by Equation (6) is the maximum. The outputs of all other neurons are considered to be 0.

The training of the PRN is accomplished via backpropagation learning algorithm [70,71,72]. The feature vectors of the CXR images and their corresponding attributes (i.e., COVID-19, normal, lung opacity, and viral pneumonia) are used as the known input pairs. Once the process of training is over, the trained PRN is tested for the feature vectors of unknown CXR images to determine their attributes. In this work, we have analyzed the classification performances of PRN for six different training algorithms as listed in Table 3.

#### 2.3.2. Classification of CXR Images Using SVM Classifier

SVM is a popular supervised learning algorithm widely used for data classification in machine learning [73,74,75]. The ultimate function of SVM in data classification applications is to create the best decision boundary, called hyperplane that facilitates the classification among different classes. The utilization of hyperplane performs well for linearly separable data [73,74]. In case of linearly inseparable data classification, SVM essentially utilizes a method called kernel trick by which the linearly inseparable data are transformed into linearly separable data to be classified by a linear classifier. In this study, Gaussian radial basis function (RBF) has been employed as the SVM kernel as it provides better performances in many machine-learning applications [75,76]. Such RBF is defined by [76]:

(7) $$\kappa \left(x,x_{c}\right)=\exp\left(-\frac{\left\|x-x_{c}{}^{2}\right\|}{2\sigma^{2}}\right).$$

**Figure 6:**
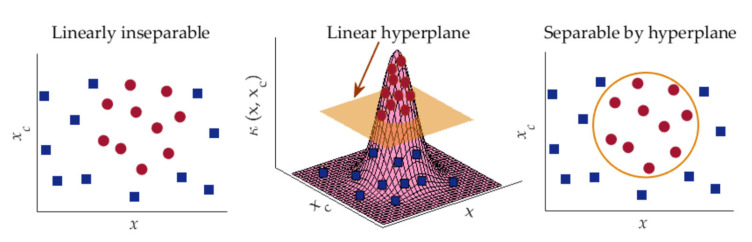
Data classification approach of SVM using kernel trick.

In Equation (7), $\left\|x-x_{c}\right\|$ is the Euclidean distance of *x* from the center *x_c_* of the function and the parameter σ controls the smoothness of the function. Since the value of *κ* decreases with the increase in Euclidean distance, the use of Equation (7) can approximate the local characteristics of a nonlinear function closed to *x_c_*. The RBF kernel nonlinearly projects two-dimensional original features onto a three-dimensional space. As a result, the linearly inseparable data can be separated by using an appropriate hyperplane. The data grouping approach of SVM via RBF kernel is depicted in Figure 6.

In SVM, the known input pairs are employed for the optimization of parameters of classification model. This optimized model is then applied to classify unknown samples. Since SVM can match various data groups acquired from training phase, it can identify images with same categories.

#### 2.3.3. Classification of CXR Images Using DT Classifier

**Figure 7:**
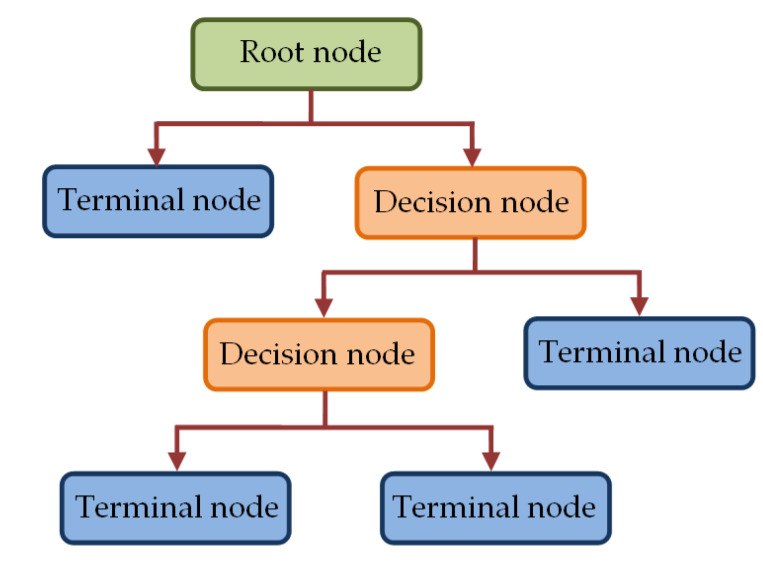
The tree structure in DT classifier.

The DT classifier used in this study is based on classification and regression tree (CART) algorithm that employs binary tree structure [77,78,79,80]. In such CART-based DT classifier, the tree structure consists of nodes, which are linked via branches as depicted in Figure 7.

The topmost node in Figure 7, called the root node, utilizes all samples to be classified based on various features to create sub-groups. A sub-group is further split in the decision node to create more sub-groups to be split by other decision nodes. Alternatively, such splitting results in final nodes (terminal nodes), which represent the label of the class. The grouping of all samples in the root node and that in the decision nodes is performed based on a predefined criterion. In our study, we have utilized Gini index criterion to construct the trees. For a group of samples *D* having *c* classes, such Gini index is calculated by [79].

(8) $$G\left(D\right)=1-\overset{c}{\underset{i=1}{\sum }}{\left(P_{i}\right)}^{2}$$

where *P_i_* corresponds to the probability of class *i* = 1, 2, …., *c* in *D*.

#### 2.3.4. Classification of CXR Images Using RF Classifier

**Figure 8:**
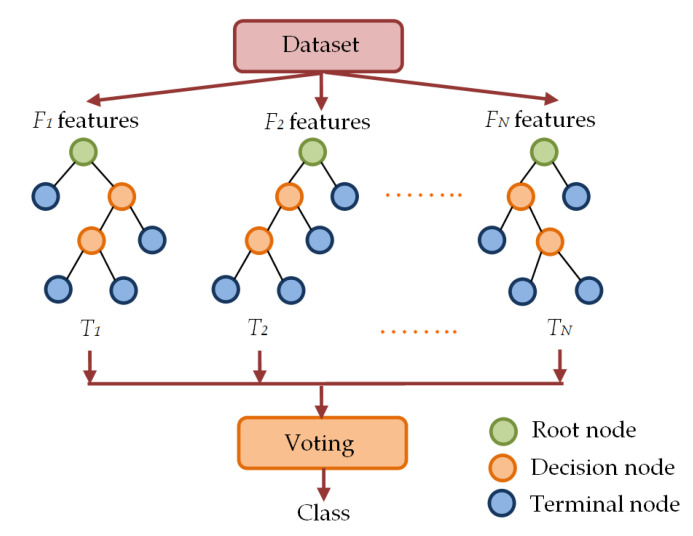
Multiple decision tree-based forest-like structure in RF classifier.

Random forest classifiers utilize multiple decision trees to form forest-like structures by employing randomly selected subsets of features from the feature set of the samples [78,80]. In this ensemble learning procedure, multiple DT-based classifiers are fitted on different subsets of features. Each of the different trees in RF classifier provides the label of a class. The class labels provided by individual trees go through a voting process and the label obtained through the majority voting is considered the final class label. The working principle of a generalized RF classifier is illustrated in Figure 8.

As shown in Figure 8, the RF classifier randomly picks subsets of features to construct random decision trees *T_1_*, *T_2_*,…, *T_N_* with corresponding labels *L_i_* of class *i* = 1, 2, …., *c*. Then, the process of majority voting is utilized to determine the final class label *L* of the RF classifier.

#### 2.3.5. Classification of CXR Images Using KNN Classifier

**Figure 9:**
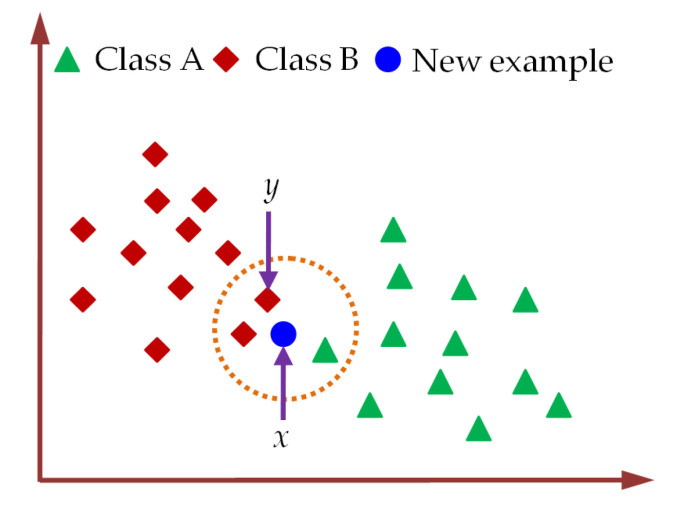
The assigning of class label to a new test sample using KNN classifier.

The KNN is a non-parametric machine learning classifier that utilizes the similarity between the available training samples and the new sample to be classified [80,81,82]. The class labels for KNN classifier are determined by calculating the closeness among each test sample and the training samples in *n*-dimensional space. The classification of a new test sample using KNN classifier is illustrated in Figure 9.

For the classification of the new test sample using KNN classifier, the number (k) of neighbors is fixed first. Then, the Euclidean distance between the new test sample and training samples is determined by Equation (9) to select k-nearest neighbors [82]. Next, the number of training samples belonging to a particular category is counted among these k-nearest neighbors. Finally, the label of the new test sample is assigned to the class for which the number of neighbors among these k-nearest neighbors is maximum. For k = 3, the new example sample in Figure 9 is categorized as class B by KNN classifiers. In practical applications of KNN classifiers, k is usually selected to be an odd number which can minimally be k = 1 [82].

(9) $$d_{E}=\overset{n}{\underset{i=1}{\sum }}{\left(x_{i}-y_{i}\right)}^{2}$$

#### 2.3.6. Classification of CXR Images Using Ensemble-CNN Based SVM Classifier

**Figure 10:**
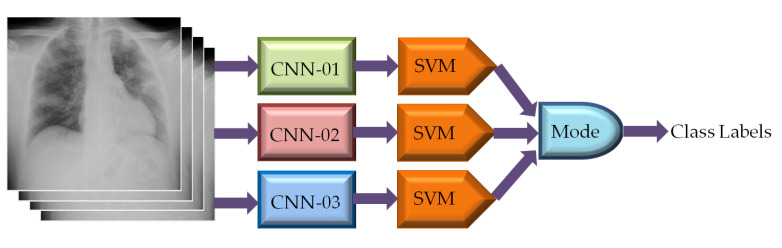
Classification of CXR images using ensemble-CNN-based SVM classifier.

Ensemble technique combines diverse models together to yield better performance than any of the constituent models. Although CNN based classifiers offer good classification performance, they can also be utilized in ensemble configuration to build robust and highly reliable classification model [83,84,85,86]. In this study, we have explored the performance of CNN-based SVMs in such ensemble configuration to further improve the classification accuracy in CXR image classification. To do so, we have identified 3 best CNNs among nineteen different pre-trained CNNs listed in Table 2 based on their classification performances. The CXR images have been applied directly to each of the 3 different CNNs for extracting feature vectors. Three SVMs are then utilized separately to classify the CXR images based on the extracted feature vectors. The mode statistics of SVMs derived CXR image class labels were considered as the ultimate image class as depicted in Figure 10.

### 2.4. Performance Evaluation of Classifiers

**Figure 11:**
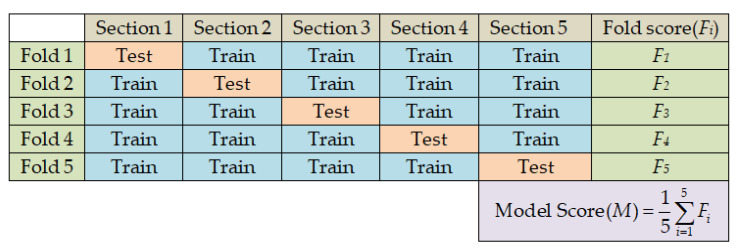
Data splitting and performance evaluation processes in five-fold cross-validation.

In this study, we have adopted five-fold cross-validation to generalize the performance of the classifiers. Each of the five folds contains different combinations of 4288 (i.e., 1072 from each class) CXR images for training and 1072 (i.e., 268 from each class) CXR images for testing the classifier as listed in Table 1. The processes of data splitting for five-fold cross-validation and performance evaluation of classifiers are illustrated in Figure 11.

To evaluate the performance of the classifiers, we first computed the performance score per-class in a particular fold. For this, four confusion matrix parameters *TP* (true positive), *TN* (true negative), *FP* (false positive), and *FN* (false negative) were estimated to calculate the four performance scores, namely, accuracy, precision, recall, and specificity by applying Equations (10)–(13) for each of the four different image classes of COVID-19, normal, lung opacity, and viral pneumonia in case of a particular fold.

(10) $$\mathrm{Accuracy}=\frac{TP+TN}{TP+FP+FN+TN}\times 100\left(\%\right)$$

(11) $$\mathrm{Precision}=\frac{TP}{TP+FP}\times 100\left(\%\right)$$

(12) $$\mathrm{Recall}=\frac{TP}{TP+FN}\times 100\left(\%\right)$$

(13) $$\mathrm{Specificity}=\frac{TN}{TN+FP}\times 100\left(\%\right)$$

The per-fold performance scores stem from the average over the four-class performances. The ultimate performance score is the average obtained from the five-fold performances. In addition to four performance metrics (i.e., accuracy, precision, recall, and specificity), the ultimate performances of the classifiers have also been assessed in terms of F1 score as given by Equation (14), in which the precision and the recall scores used are the ultimate performance scores of the classifier.

(14) $$F1\;\mathrm{score}=2\times \frac{\mathrm{Precision}\times \mathrm{Recall}}{\mathrm{Precision}+\mathrm{Recall}}\times 100\left(\%\right)$$

In this study, we have extracted the image features of the training images offline. The classifiers have also been trained on the extracted features offline during the training phase. However, the feature extraction and classification of different trained classifiers in the testing phase are performed online to facilitate comparative analysis of their runtime. The whole system was implemented by MATLAB R2021a on a workstation with Intel^®^ Core™ i7-117000@2.50 GHz, 8 cores 16 logical processors, HP Ex900 M.2 500 GB PCIe NVMe Internal SSD, Gigabyte GeForce RTX 2060 OC 6 GB Graphics card, and 16 GB DDR4 RAM memory.

## 3. Results and Discussion

**Figure 12:**
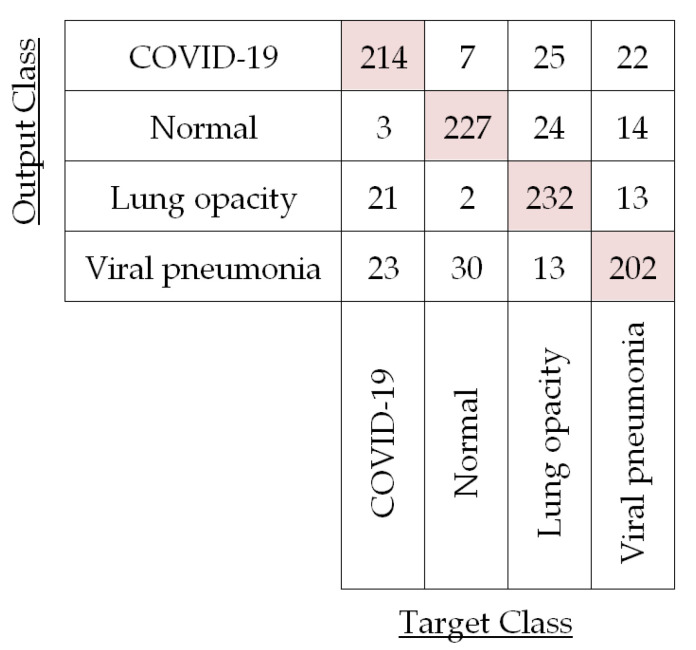
Confusion matrix for the test CXR images in first fold (Fold 1) using LBP-based SVM.

In our demonstration of four-class (i.e., COVID-19, normal, lung opacity, and viral pneumonia) classification, we have extracted the feature vectors of CXR images by using the LBP operator as well as pre-trained CNNs. Then, the classification has been performed by PRN, SVM, DT, RF, and KNN classifiers based on those extracted feature vectors. We have also evaluated the classification performances of LBP-based PRN, LBP-based SVM, LBP-based DT, LBP-based RF, LBP-based KNN, CNN-based SVM, and ensemble-CNN-based SVM by adopting the process of five-fold cross-validation as depicted in Figure 11. For instance, the confusion matrix for the testing images in a particular fold (i.e., Fold 1) is shown in Figure 12 for LBP-based SVM.

The confusion matrix in Figure 12 corroborates LBP-based classifiers’ success and is used to compute the performances of LBP-based SVM for each of the four different classes in Fold 1 by using Equations (10)–(13) as shown in Table 4.

It is observed in Table 4 that per-class classification accuracy for the test CXR images of each of the four different classes is around 90%. Now, we have averaged these per-class accuracies obtained for this fold to compute fold accuracy. For this Fold 1, LBP-based SVM yields 90.81% accuracy. We have also calculated the fold precision (81.62%), fold recall (81.68%), and fold specificity (93.90%) by averaging per-class precision, recall, and specificity, respectively, for Fold 1. In a similar fashion, we have calculated the per-fold accuracy, precision, recall, and specificity for the other four folds in the case of LBP-based SVM. The results for each of the five folds are listed in Table 5.

The overall performance of this classification model is computed by averaging five per-fold performances as listed in Table 5. Consequently, the overall accuracy, precision, recall, and specificity for using LBP-based SVM have been found to be 88.86%, 77.72%, 79.80%, and 92.58%, respectively. The overall F1 score for LBP-based SVM classification turned out to be 78.75% following Equation (14).

**Figure 13:**
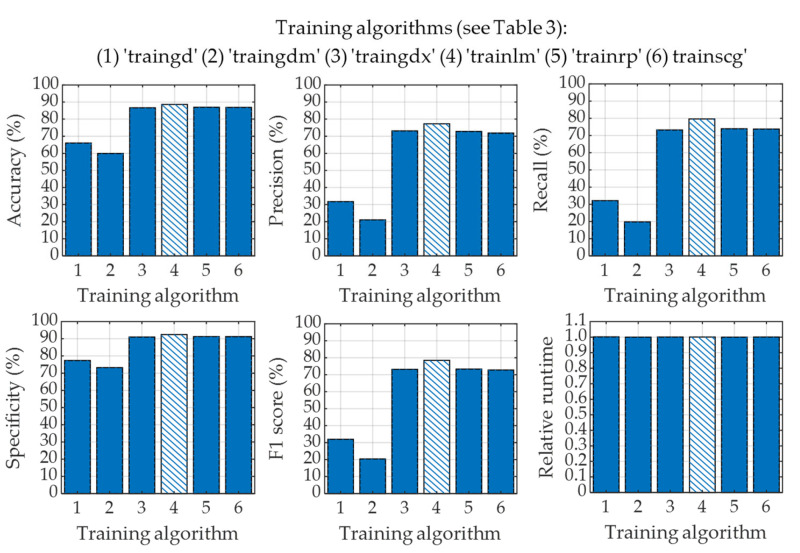
Classification performance for using different training algorithms in LBP-based PRN.

Next, we have applied a pattern recognition network (PRN) to classify four classes of CXR images utilizing the image feature vectors obtained from the LBP operator. In this classification process, we have also employed five-fold cross-validation to generalize the overall performance of LBP-based PRN. In this study, we have also explored the effects of applying six different training algorithms to train the PRN as listed in Table 3. The overall classification performances of LBP-based PRN for adopting each of the six training algorithms are shown in Figure 13.

The performances of LBP-based PRN vary with training algorithms used to train the PRN as seen in Figure 13. It is evident that gradient descent (“traingd”) and gradient descent with momentum (“traingdm”) training algorithms failed to perform well when used with LBP-based PRN. It can also be observed in Figure 13 that the performances of LBP-based PRN are comparable if such PRN is trained with variable learning rate gradient descent (“traingdx”), Levenberg–Marquardt (“trainlm”), resilient backpropagation (“trainrp”) and scaled conjugate gradient (“trainscg”) learning algorithms. However, the training of LBP-based PRN by adopting the Levenberg–Marquardt algorithm provides the best performances with accuracy, precision, recall, specificity, and F1 score of 88.61%, 77.28%, 79.60%, 92.44%, and 78.42%, respectively. It is worth mentioning that all six different algorithms mentioned in Figure 13 have very similar runtime in the testing phase.

In a similar fashion, we have determined the classification performances of LBP-based DT, LBP-based RF, and LBP-based KNN. For instance, the overall accuracies provided by LBP-based DT, LBP-based RF, and LBP-based KNN are computed to be 83.77%, 87.43%, and 84.58%, respectively. The SVM yields the best accuracy in all cases of LBP-based machine learning classifiers used in this study. Consequently, we have only considered the SVM classifier for classifying CXR images in the next stage.

**Figure 14:**
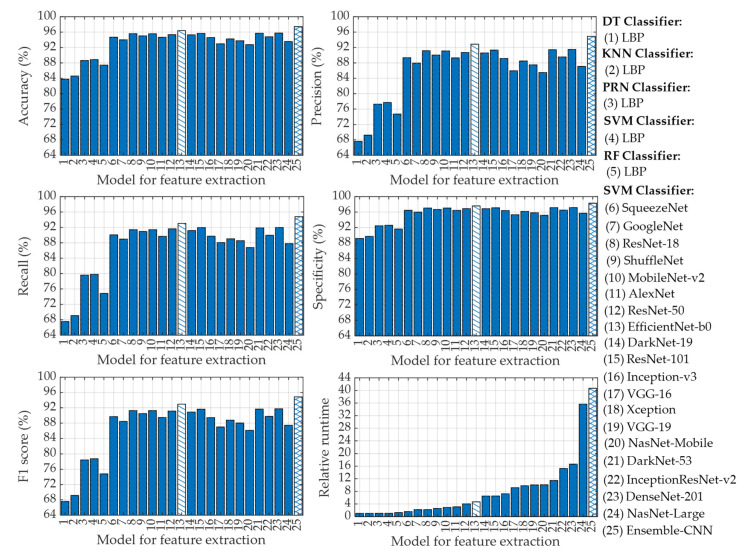
Classification performances for using LBP-based DT, LBP-based KNN, LBP-based PRN, LBP-based SVM, LBP-based RF, CNN-based SVM, and ensemble-CNN-based SVM classifiers.

In this stage, we have extracted feature vectors from the CXR images by applying a deep learning algorithm where features are taken from the fully connected layer at the end of the CNN. To accomplish this feature extraction using CNN, we have employed a total of nineteen different pre-trained CNNs as listed in Table 2. After extracting the feature vectors from the CXR images with CNNs, we have applied the SVM classifier in this stage for the four-class classification of CXR images as described previously. In this stage, we have also applied five-fold cross-validation to generalize the performance of CNN-based SVM classifiers. The overall classification performances of CNN-based SVM for adopting each of the nineteen different pre-trained CNNs along with the LBP-based different machine learning classifiers are shown in Figure 14.

It is easily observed in Figure 14 that each of the nineteen CNN-based SVM classifiers outperforms LBP-based different classifiers (i.e., DT, KNN, PRN, SVM, and RF). This is due to the fact that CNNs utilize a deep learning algorithm that is extremely powerful to extract feature vectors from the CXR images [60,61,62,63]. For instance, the lowest accuracy in Figure 14 provided by the pre-trained CNN model of the NasNet-Mobile-based SVM is 92.74%, which is even better than that of LBP-based DT (83.77%), LBP-based KNN (84.58%), LBP-based PRN (88.61%), LBP-based SVM (88.86%), and LBP-based RF (87.43%) classifiers. However, the classification performances of SVM utilizing image feature vectors extracted with EfficientNet-b0 (model 13 in Figure 14) are the best among the nineteen pre-trained CNN architectures as can be seen from Figure 14. Such “EfficientNet-b0” pre-trained CNN-based SVM can achieve overall accuracy, precision, recall, specificity, and F1 score of 96.39%, 92.86%, 93.04%, 97.59%, and 92.95%, respectively.

To further improve the classification performance of CNN-based SVM, we have finally utilized ensemble-CNN-based SVM as described in Section 2.3.6. To effectively utilize such ensemble configuration, we have selected the best three pre-trained CNNs (i.e., EfficientNet-b0, DenseNet-201, and DarkNet-53) among the nineteen different CNN architectures used in this study based on their classification metrics. The classification performances of ensemble-CNN-based SVM have also been plotted in Figure 14 (model 25) for the purpose of comparison. The topmost classification performances provided by different feature extraction-based classifiers are listed in Table 6.

It is seen in Table 6 that the classification performances attained for using ensemble-CNN-based SVM are the highest among the 25 different classifiers adopted in this study. For instance, the ensemble-CNN-based SVM can improve the classification accuracy by ~1% as compared to the best CNN-based SVM (i.e., EfficientNet-b0-based SVM). These overall performances of EfficientNet-b0-based SVM and ensemble-CNN-based SVM are more promising compared to some recently published results as listed in Table 7.

As observed in Table 7, the performances attained by applying an Efficient-b0-based SVM classifier are much better than that achieved in Ref. [60] for four-class classification using 1251 CXR images. The overall accuracy of the Efficient-b0-based SVM classifier is also comparable to that achieved in Ref. [64]. It is to be noted that the dataset used in Ref. [64] is imbalanced as there is a big difference in the number of images in each of the four classes (with only 68 CXR images in the COVID-19 class). However, the ensemble-CNN-based SVM classifier used in this study for four-class classification can provide much-improved classification performances as compared to other methods listed in Table 7 with overall accuracy, precision, recall, specificity, and F1 score of 97.41%, 94.91%, 94.81%, 98.27%, and 94.86%, respectively. To the best of our knowledge, these classification performances rank the best among all other reported values for four-class classification of COVID-19, normal, lung opacity, and viral pneumonia CXR images in the existing literature.

Now we focus on relative runtime comparison analysis among different classifiers that use different feature extraction algorithms as shown in Figure 14. It is evident that LBP-based DT requires the lowest runtime. Thus, the relative runtime of a particular technique is normalized with respect to the runtime taken by LBP-based DT. The relative runtimes of different LBP-based machine learning classifiers are nearly uniform with LBP-based RF being the slowest. However, the relative runtimes of CNN-based SVMs vary in accordance with the depth of the layered architecture of the pre-trained CNNs. Among them, the SqueezeNet-based SVM yields the lowest runtime while the NasNet-Large-based SVM requires the highest relative runtime as can be seen in Figure 14. However, the relative runtime of the single CNN (i.e., EfficieNet-b0)-based SVM which provides the best classification performances is moderately low as compared to other CNN architectures used in this study. To be specific, the “EfficieNet-b0”-based SVM is 4.63 times slower as compared to LBP-based DT. However, such “EfficieNet-b0”-based SVM can provide significantly improved classification performances compared to that of LBP-based machine learning classifiers as shown in Figure 14. It is also observed in Figure 14 and Table 6 that the ensemble-CNNs-based SVM provides the highest classification performance (e.g., 97.41% accuracy) among all the classifiers used in this study. However, to achieve such high performance, this classifier requires relative runtime of 40.68 (i.e., 40.68 times higher than LBP-based DT), which is ~8.72 times larger than that of “EfficieNet-b0”-based SVM.

## 4. Conclusions

This paper presents a rigorous study on the identification of COVID-19 infection from CXR images based on machine learning approaches. The feature vectors of CXR images have been extracted successfully by utilizing LBP operator and pre-trained CNNs of nineteen different architectures. Then, PRN, SVM, DT, RF, and KNN classifiers have been applied to classify four-class CXR images comprising COVID-19, normal, lung opacity, and viral pneumonia by utilizing the extracted feature vectors of the CXR images. The performances of LBP-based PRN, LBP-based SVM, LBP-based DT, LBP-based RF, LBP-based KNN, CNN-based SVM, and ensemble-CNN-based SVM classifiers have been investigated in detail on the four-class test images and their performances are analyzed in terms of accuracy, precision, recall, specificity, F1 score, and relative runtime. The effects of using six different learning algorithms used to train the LBP-based PRN are analyzed in detail and the results indicate that the Levenberg–Marquardt learning algorithm provides the best classification performance for using LBP-based PRNs in this study. The results also show that the classification performances of LBP-based classifiers are not up to the mark and are significantly lower than that of CNN-based SVM. Among nineteen different single pre-trained CNN-based SVM classifiers, the use of EfficientNet-b0 CNN architecture performs best in our study. The use of such CNN architecture can achieve overall classification performances of 96.39% accuracy, 92.86% precision, 93.04% recall, 97.59% specificity, and 92.95% F1 score with moderately low relative runtime. To further improve the classification performance of CNN-based SVM, we have also utilized ensemble-CNN-based SVM. Such an ensemble configuration consisting of three pre-trained CNNs (i.e., EfficientNet-b0, DenseNet-201, and DarkNet-53) has provided improved classification performances with 97.41% accuracy, 94.91% precision, 94.81% recall, 98.27% specificity, and 94.86% F1 score but required highest runtime to classify CXR images. We believe that the strategy suggested in this paper will provide doctors and physicians with a complementary tool for the diagnosis and prognosis of COVID-19-infected patients. Moreover, the framework so proposed can be integrated into a decision support system that can diagnose COVID-19 based on CXR images, thus considerably minimizing both human and machine error.

## Figures and Tables

**Table 1:** Total number of chest X-ray images per-class and per-fold.

Class	Number of Total CXR Images			
	Dataset	Used	Training per-Fold	Testing per-Fold
COVID-19	3616	1340	1072	268
Normal	10,192	1340	1072	268
Lung opacity	6012	1340	1072	268
Viral pneumonia	1345	1340	1072	268

**Table 2:** Feature layer of different pre-trained CNNs.

Pre-Trained CNN	Name of the Feature Layer
SqueezeNet	“pool10”
GoogleNet	“loss3-classifier”
ResNet-18	“fc1000”
ShuffleNet	“node202”
MobileNet-v2	“Logits”
AlexNet	“fc8”
ResNet-50	“fc1000”
EfficientNet-b0	“efficientnet-b0|model|head|dense|MatMul”
DarkNet-19	“avg1”
ResNet-101	“fc1000”
Inception-v3	“predictions”
VGG-16	“fc8”
Xception	“predictions”
VGG-19	“fc8”
NasNet-Mobile	“predictions”
DarkNet-53	“conv53”
InceptionResNet-v2	“predictions”
DenseNet-201	“fc1000”
NasNet-Large	“predictions”

**Table 3:** Training algorithms used to train the PRN classifier.

Training Algorithm	Matlab Function
Gradient Descent (GD)	“traingd”
GD with Momentum	“traingdm”
Variable Learning Rate GD	“traingdx”
Levenberg–Marquardt	“trainlm”
Resilient Backpropagation	“trainrp”
Scaled Conjugate Gradient	“trainscg”

**Table 4:** Classification performance for the test CXR images in Fold 1 using LBP-based SVM.

Class	Accuracy (%)	Precision (%)	Recall (%)	Specificity (%)
COVID-19	90.58	79.85	81.99	93.34
Normal	92.54	84.70	85.34	94.91
Lung opacity	90.86	86.57	78.91	95.37
Viral pneumonia	89.27	75.37	80.48	91.96
**Average**	**90.81**	**81.62**	**81.68**	**93.90**

**Table 5:** Classification performance per-fold for the test CXR images using LBP-based SVM.

Fold	Accuracy (%)	Precision (%)	Recall (%)	Specificity (%)
1	90.81	81.62	81.68	93.90
2	90.30	80.60	80.77	93.53
3	91.09	82.18	82.16	94.06
4	90.86	81.72	81.82	93.91
5	81.25	62.50	72.59	87.50
**Average**	**88.86**	**77.72**	**79.80**	**92.58**

**Table 6:** Topmost classification performance provided by different classifiers.

Classifier	Accuracy (%)	Precision (%)	Recall (%)	Specificity (%)	F1 Score (%)
LBP + DT	83.77	67.62	67.54	89.18	67.58
LBP + KNN	84.58	69.22	69.08	89.72	69.15
LBP + RF	87.43	74.70	74.85	91.62	74.77
LBP + PRN (“trainlm”)	88.61	77.28	79.60	92.44	78.42
LBP + SVM	88.86	77.72	79.80	92.58	78.75
DarkNet-53 + SVM	95.72	91.44	91.88	97.15	91.66
DenseNet-201 + SVM	95.76	91.51	91.96	97.17	91.74
EfficientNet-b0 + SVM	96.39	92.86	93.04	97.59	92.95
**Ensemble-CNN + SVM ^1^**	**97.41**	**94.91**	**94.81**	**98.27**	**94.86**

^1^ Ensemble configuration of EfficientNet-b0, DenseNet-201, and DarkNet-53 pre-trained CNNs

**Table 7:** Comparison of recently published studies on COVID-19 detection from CXR images **^1^**.

Authors	Methods	Total Images	Classes	A (%)	P (%)	R (%)	S (%)	F1 (%)
Vinod et al. [45]	CNN + DT	300	2	-	88.00	83.00	-	85.00
Ozturk et al. [40]	DarkCovidNet	125	2	98.08	98.03	95.13	95.3	96.51
Wang et al. [22]	COVID-Net	13,962	3	83.50	-	91.00	-	-
Heidari et al. [56]	CNN + CAD	8474	3	94.50	98.40	-	98.00	94.00
Vinod et al. [58]	Covix-Net	9000	3	96.80	98.00	99.00	-	-
Khan et al. [60]	CoroNet	1251	4	89.60	93.00	98.20	96.40	95.60
Farooq et al. [64]	COVID-ResNet	2862	4	96.23	-	-	-	-
**This paper**	**EfficientNet-b0 + SVM**	**5360**	**4**	**96.39**	**92.86**	**93.04**	**97.59**	**92.95**
**This Paper**	**Ensemble-CNN + SVM**	**5360**	**4**	**97.41**	**94.91**	**94.81**	**98.27**	**94.86**

^1^ A: Accuracy, P: Precision, R: Recall, S: Specificity, F1: F1 score.

## Data Availability

The dataset used in this study is available at the following link: https://www.kaggle.com/datasets/tawsifurrahman/covid19-radiography-database (accessed on 12 May 2022).
